# A new ether-based electrolyte for dendrite-free lithium-metal based rechargeable batteries

**DOI:** 10.1038/srep21771

**Published:** 2016-02-16

**Authors:** Rongrong Miao, Jun Yang, Zhixin Xu, Jiulin Wang, Yanna Nuli, Limin Sun

**Affiliations:** 1 School of Chemistry and Chemical Engineering, Shanghai Jiao Tong University, 800 Dongchuan Road, Shanghai, 200240, China; 2 Instrumental Analysis Center, Shanghai Jiao Tong University, Shanghai 200240, P. R. China

## Abstract

A new ether-based electrolyte to match lithium metal electrode is prepared by introducing 1, 4-dioxane as co-solvent into lithium bis(fluorosulfonyl)imide/1,2-dimethoxyethane solution. Under the synergetic effect of solvents and salt, this simple liquid electrolyte presents stable Li cycling with dendrite-free Li deposition even at relatively high current rate, high coulombic efficiency of *ca.* 98%, and good anodic stability up to ~4.87 V vs Li RE. Its excellent performance will open up a new possibility for high energy-density rechargeable Li metal battery system.

Lithium metal has been regarded as an ideal anode in rechargeable lithium battery systems over the past four decades because of its high theoretical specific capacity (3860 mAh g^−1^) and the most negative electrochemical potential(−3V vs. standard hydrogen electrode, SHE) among anode materials^1^,^2^,^3^. The increasing demand for various electric vehicles has motivated the development of high-energy storage systems, in which the use of lithium metal anode can lead to as much as a tenfold improvement in anode storage capacity compared with carbon anode(~360 mAh g^−1^) in traditional Li-ion batteries and would open up opportunities for high-energy unlithiated cathode materials such as the highly promising sulphur and oxygen^4^,^5^. However, the formation of dendritic lithium and low coulombic efficiency during repeated charge/discharge cycles have hindered its practical application in rechargeable lithium-metal based batteries^6^. Particularly, Li dendrite and “dead” Li thus created could lead to serious safety hazards including thermal runaway or even fire/explosion of the battery.

Generally, uncontrolled growth of lithium dendrites is mainly induced by inhomogeneous distributions of (1) the current density on the electrode surface, (2) the concentration gradient of Li ions at the electrolyte/electrode interface, and (3) the solid-electrolyte interphase (SEI) layer^7^. The formation of a SEI layer with high uniformity and stability is essential to ensure high coulombic efficiency, long cycle life and safety in lithium-metal based batteries^3^. Among various approaches to address the issues of lithium metal anode, it has long been recognized that electrolyte selection is one of the most critical factors that affects the cycling stability of Li metal anodes. Although the use of solid-state electrolytes appears to potentially suppress the growth of lithium dendrites and essentially improve the battery safety, the limited kinetic properties resulted from low conductivity at room temperature and high interfacial resistance are the persistent obstacles for its application^8^,^9^. Moreover, based on the viewpoint proposed by Zhang *et al*.^10^, the solid polymer actually serves as an effective physical barrier to block dendrite penetration, but the growth mechanism of Li dendrites has not been altered on a fundamental level. Consequently, dendritic or mossy Li may still be generated beneath the physical barrier and lead to cell failure. Of liquid electrolytes, organic carbonate-based electrolytes are commonly employed in Li-ion batteries with carbon anode for its acceptable anodic stability. However, it is well established that the plating/stripping performance of Li metal in electrolytes with carbonate solvents such as propylene carbonate (PC) is poor and typically results in dendritic Li metal deposits and a low coulombic efficiency^11^. Thus, many additives including organic compounds (e.g. 2-methylfurane, 2-methylthiophene, etc^12^,^13^.), inorganic ions (e.g. Al^3+^, Mg^2+^, I^−^, etc^14^,^15^.), active gases (e.g. SO_2_^16^, CO_2_^17^) or recently proposed halogenated salt^18^ (e.g. LiF or LiBr) have been employed to improve the uniformity of SEI layer and further suppress the dendritic lithium deposition^3^,^16^,^18^,^19^,^20^,^21^. In addition to the individual additive, some hybrid additives, such as the combination of lithium polysulfide and lithium nitrate, are developed as well^3^. Although these strategies are promising to some extent, their effectiveness in suppressing dendrite growth may be undermined by consumption of additives during the formation of the SEI films over successive charge−discharge cycles, especially at high current densities^22^. Lately, Xu *et al*. proposed a carbonate electrolyte with CsPF_6_ as additive, which can prevent the dendrite formation without its consumption under the self-healing electrostatic shielding mechanism^10^. Nevertheless, its effectiveness may be diminished at large current density because of the co-deposition with lithium^6^. Moreover, when carbonate based electrolyte was used in Li-O_2_ batteries, instead of O_2_ being reduced in the porous cathode to form Li_2_O_2_ as desired, the carbonate based electrolyte always undergoes severe decomposition during discharging process^23^,^24^. Thereby, the exploration of new liquid electrolyte formulations, as the most simple approach to practical applications, with good compatibility with lithium and high current density tolerance is critically important.

Previous research of screened various solvents in the liquid electrolyte system suggests that ether-based electrolytes, commonly used in high-energy lithium–sulfur and lithium–air battery systems, exhibit much better performance due to the formation of flexible oligomers in SEI layers on lithium anode^3^,^25^. To date, the most predominant ether-solvents used for Li metal anode are THF, DOL, DME or their combination. However, the poor anti-oxidation capability of ether-based electrolytes may hamper their wider utilization in Li-O_2_ batteries or even moderate voltage cathodes (3~4V). For instance, we developed a dual-salts electrolyte, which exhibits dendrite-free lithium deposition even at high current density of 10 mA cm^−2^, while the limited potential window of ~3.8V still needs a further improvement^26^. Most recently, the ether-based electrolyte system (4M LiFSI/DME) proposed by Zhang *et al*. exhibits much higher anodic stability of 4.5 V^27^. But the highly concentrated Li-salt commonly means a higher electrolyte cost. Even for Li-S and Li-O_2_ cells with low thermodynamic cathode reaction potentials below 3 V vs. Li/Li^+^, the high overpotentials originating from various reasons, such as poor conductivity of reaction products or ions diffusion control at large current density, might require a higher charging potential, leading to a big risk of anodic decomposition of electrolytes^28^. In fact, the polysulfides commonly generated in Li-S system can diffuse freely through separator and react with the metal lithium anode, the reaction products precipitated on lithium surface may induce the increase of impedance and overpotential^29^. In a Li-O_2_ cell system, practical charging voltage often reaches 4 V or even higher. Therefore, it is significant to explore new ether-based electrolytes with a wider anodic window, good compatibility with lithium and appropriate cost.

Herein we develop a new and simple ether-based electrolyte composed of 1, 4-dioxane (DX) and 1,2-dimethoxyethane (DME) as ether-solvents and lithium bis(fluorosulfonyl)imide (LiFSI or LiN(SO_2_F)_2_) as lithium salt. Its compatibility to Li metal electrode and the electrochemical behaviour of rechargeable batteries based on Li metal anode and this electrolyte are investigated. The related function mechanism is discussed as well.

## Results

### Cyclic voltammograms

**Figure 1:**
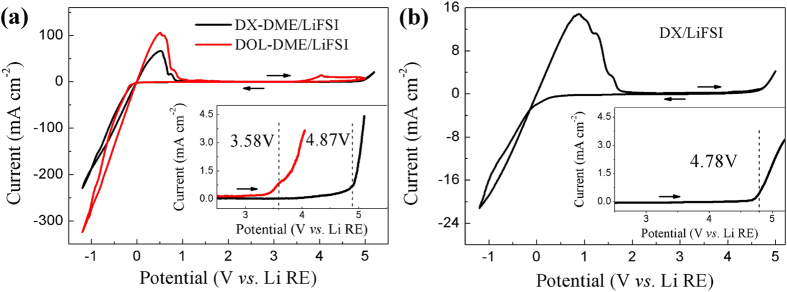
Steady-state cyclic voltammograms for the first cycle on Pt electrode with different electrolytes. (**a**) DX-DME(1:2)/1M LiFSI and DOL-DME (1:1)/1M LiFSI electrolytes at a scanning rate of 20 mV s^−1^, inset is enlarged anodic region at scanning rate of 1 mV s^−1^; (**b**) DX/1M LiFSI electrolyte at a scanning rate of 20 mV s^−1^, inset is enlarged anodic region at scanning rate of 1 mV s^−1^.

Although it has been well established that the theoretical electrochemical stability of a electrolyte is defined by its difference between LUMO and HOMO from a thermodynamic stability standpoint, the coupling effects of solvent/salt on electrolyte stability is noteworthy as well^30^. Thereby, to ascertain the accessible electrochemical window of all electrolytes under study, cyclic voltammetry (CV) was conducted by using a three-electrode cell. As shown in Fig. 1a, the electrolyte solution containing DX as co-solvent is electrochemically stable to ~4.87 V vs. Li^+^/Li on Pt, well above that of generally used DOL-DME solution (~3.58 V). Compared with the lately reported concentrated DME/4M LiFSI solution (oxidation potential ~4.5 V), higher oxidative stability can be obtained with dilute salt concentration. The neat DX based solution as shown in Fig. 1b exhibits high stability of ~4.78 V as well, which implies good oxidation-resistant capability of DX. The good anodic stability of DX-DME/LiFSI solution widens its usage in lithium-metal based batteries.

It should be metioned that additional small oxidation peaks near Li stripping can be observed in the first cycle, which might be caused by electrocatalytic reactions of SEI film via direct Pt contact. When replacing Pt electrode by stainless steel (SS) electrode, there is no such oxidation peaks appeared in both the electrolytes (Supplementary Fig. S1).

### Coulombic efficiency

**Figure 2:**
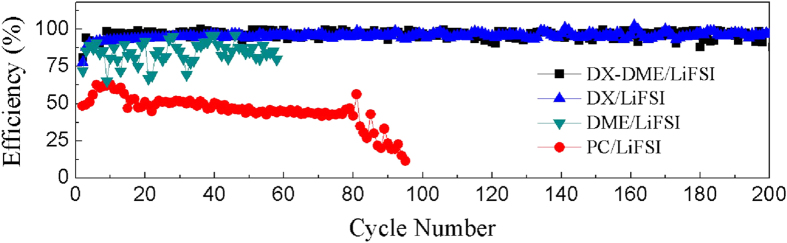
Coulombic efficiency of Li-SS cells cycled in different electrolyte solutions. Each dissolution and deposition of lithium-metal was performed at 25 °C under constant deposition current of 0.25 mA cm^−2^ for 2.5 h (*ca*. 4.5C), and then the same dissolution current density was applied until the cut-off voltage of 1.2 V vs. Li.

In order to examine the coulombic efficiency of lithium deposition/dissolution processes, galvanostatic cycling experiments were conducted. The efficiency was calculated according to the ratio of the charge amount of complete Li dissolution to that of Li deposition on SS. It can be observed from Fig. 2 that Li-SS cell can be cycled for 200 cycles with a stable and high coulombic efficiency of ~98% in DX-DME/LiFSI solution, which is advantageous over others without DX or with PC as solvent. The similar coulombic efficiency in DX/LiFSI solution further indicates the good compatibility of DX solvent with lithium. The higher coulombic efficiency of DX containing electrolyte may be associated with its extremely low reduction potential (−1.95 V vs Li/Li^+^, even lower than that of DME: −1.68 V vs Li/Li^+^), calculated according to *ab initio* DFT^25^, which implies a low tendency of a direct reaction between DX solvent and Li metal. The high cycle stability and electrochemical reversibility of lithium deposition/dissolution in the electrolytes containing DX solvent provides a new possibility for lithium metal anode used in nonaqueous liquid electrolyte.

### Observation of Li morphologies deposited in different electrolytes

**Figure 3:**
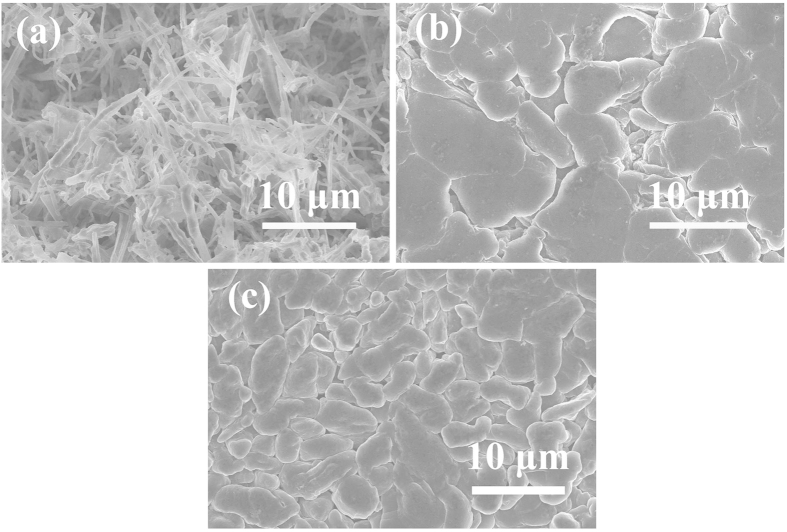
SEM images of Li metal after plating on Cu substrates in different electrolytes. (**a**) PC/1M LiFSI; (**b**) DX/1M LiFSI; (**c**) DX-DME(1:2)/1M LiFSI; The current density was 0.25 mA cm^−2^ and the deposition time was 6 h (~5.4C cm^−2^). The diameter of the Cu substrate was 12 mm.

The morphologies of Li deposition in different electrolytes were evaluated by using coin-type Cu|Li cells. A fixed amount of lithium (~ 5.4 C cm^−2^) was firstly deposited onto the copper substrate at a relatively small current density of 0.25 mA cm^−2^, followed by disassembling the coin cell to harvest the the deposited lithium on Cu substrate for subsequent microscopic analysis. Fig. 3a shows the surface morphology of the Li deposition in a carbonate-based electrolyte (1 M LiFSI in PC), where extensive Li dendrites are clearly observable. The needle-like structure with sharp-end may penetrate through separator and incur internal short circuit, resulting in safety issues. Additionally, its high surface area associated with the dendritic morphology and side reactions results in the extremely low coulombic efficiency of only ~50% (Fig. 2). In contrast, a non-dendritic Li deposition with flat and pancake-like structure was obtained in DX-LiFSI electrolytes (Fig. 3b), corresponding to a much higher and more stable coulombic efficiency (Fig. 2), which indicates that DX is helpful for forming non-dendritic lithium deposits. Based on the advantage of DX, the DX-DME/LiFSI electrolyte with much higher solubility of LiFSI is developed, which produces a uniform and dendrite-free lithium deposition with much smaller particle size of ~5μm compared with the size of ~10μm in DX/LiFSI electrolyte (Fig. 3c).

### Lithium cycle performance at high current density

**Figure 4:**
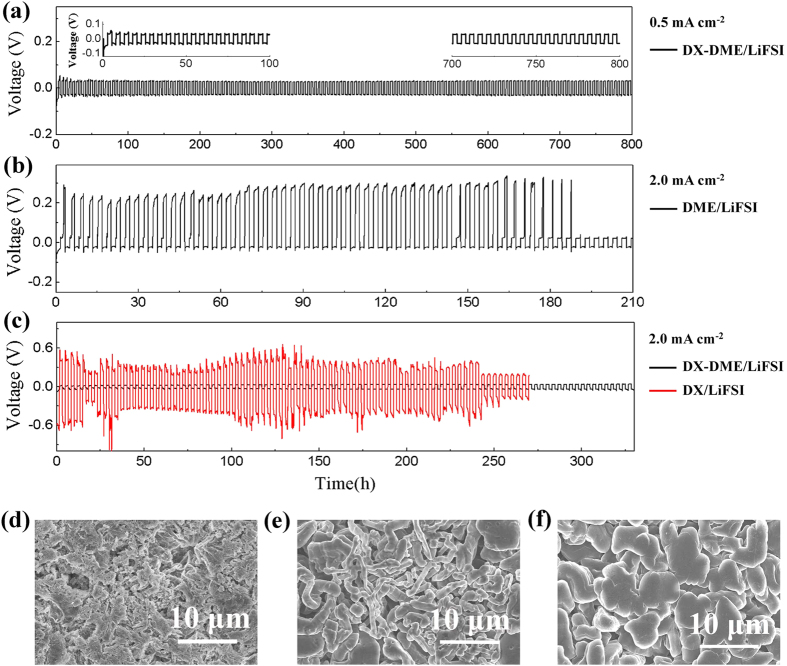
Voltage versus time curves for a symmetric lithium cell and SEM analyses. (**a**) voltage profiles for DX-DME(1:2)/1M LiFSI electrolyte at current density of 0.5 mA cm^−2^ and the inset plots are expanded views of the bottom curve; (**b**) voltage profiles for DME/1M LiFSI electrolyte at current density of 2.0 mA cm^−2^; (**c**) Typical time-dependent voltage for DX-DME(1:2)/1M LiFSI electrolyte (black) and DX/1M LiFSI electrolyte (red) at current density of 2.0 mA cm^−2^; (**d**–**f**) SEM micrographs of deposited lithium after 170h (50 cycles) of charge/discharge in DME/1M LiFSI electrolyte, DX/1M LiFSI electrolyte and DX-DME(1:2)/1M LiFSI electrolyte respectively with a current density of 2.0 mA cm^−2^. The symmetric lithium cells cycled in above conditions last 1.7 h for each half-cycle.

A symmetric cell was used to investigate the stability of the Li metal anode in different electrolytes. Figure 4a shows the long-term cycling stability of a coin-type symmetric lithium cell with the DX-DME/LiFSI electrolyte at current density of 0.5 mA cm^−2^. The cell was cycled for over 800h and still maintained extremely stable voltage polarizations, which demonstrates a high lithium plating/stripping cycling reversibility in this electrolyte. As well known, the inhibition of lithium dendrite growth at high current densities (>0.5 mA cm^−2^) remains a challenge, and is considered as a roadblock for lithium-metal batteries to reach the market place^31^. Therefore, a much more aggressive current density of 2.0 mA cm^−2^ was employed to examine the high-rate cycling performance of different electrolytes. For DME/LiFSI electrolyte, the stripping voltage drastically fluctuates with high polarization (Fig. 4b), corresponding to a mossy lithium morphology as shown in Fig. 4d. Nevertheless, an extremely stable voltage polarization (~36 mV) up to 330h’ cycling is achieved in DX-DME/LiFSI electrolyte (Fig. 4c). However, the cycling of symmetric cells using neat DX solvent electrolyte at this current density results in greatly increased voltage polarizations (over 370 mV) and random voltage oscillations. The poor electrode kinetics is not only due to its relatively low ionic conductivity of 3.43 mS cm^−1^ compared to 7.25 mS cm^−1^ for DX-DME/LiFSI electrolyte, but also to the different interfacial property. As exhibited in Fig. 4d, fibre-like lithium twined deposit is observable after 50 cycles in DX/LiFSI. In contrast, uniform lithium particles with smooth-surface are obtained in DX-DME/LiFSI at the same condition (Fig. 3f).

**Figure 5:**
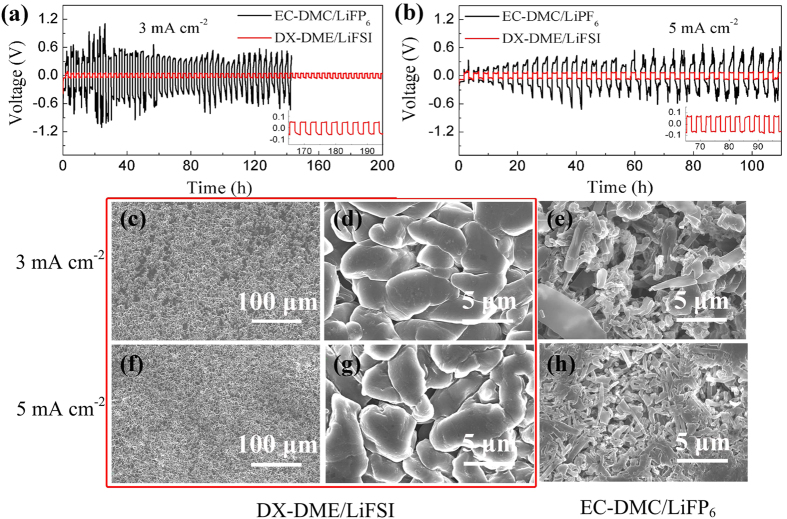
Voltage versus time curves for a symmetric lithium cell under high rate and SEM analyses. Voltage profiles for DX-DME(1:2)/1M LiFSI electrolyte and EC-DMC/1M LiPF_6_ electrolyte at (**a**) current density of 3 mA cm^−2^, (**b**) current density of 5 mA cm^−2^; SEM micrographs of deposited lithium after 170 h (50 cycles) of plating/striping process in (**c**,**d**) DX-DME/1M LiFSI electrolyte and (**e**) EC-DMC/1M LiPF_6_ electrolyte at current density of 3 mA cm^−2^; SEM micrographs of deposited lithium after ~100 h (30 cycles) of plating/striping process in (**f**,**g**) DX-DME/1M LiFSI electrolyte and (**h**) EC-DMC/1M LiPF_6_ electrolyte at current density of 5 mA cm^−2^; The symmetric lithium cells cycled in above conditions last 1.7 h for each half-cycle and final state for SEM is deposited state.

The cycling stabilities of lithium in this new ether-based electrolyte and EC-DMC/LiPF_6_ electrolyte for comparison were further examined with higher current densities of 3 mA cm^−2^ and 5 mA cm^−2^. As demonstrated by Fig. 5, the stable cycling performance (Fig. 5a,b) and uniform Li deposit composed of smooth and densely cumulated particles (5~10 μm in diameter) can be seen in DX-DME/LiFSI electrolyte after cycling at both current densities of 3 mA cm^−2^ and 5 mA cm^−2^ (Fig. 5c,d,f,g). In contrast, the large polarization and random voltage oscillations are observed under the same condition in EC-DMC/1M LiPF_6_ electrolyte (Fig. 5a,b) and accordingly a porous and needle-like deposit structure is formed (Fig. 5e,h), which may further incur cell short circuits. When the current density rises from 3 mA cm^−2^ to 5 mA cm^−2^, the voltage polarization increases mildly from ~53 mV to ~65 mV in DX-DME/LiFSI electrolyte and the overvoltage is basically stable upon time on each plating cycle (*ca*. 8.3 mAh cm^−2^). This is greatly different from the case in EC-DMC/1M LiPF_6_, where the overvoltage significantly increases with the plating time at 5 mA cm^−2^, indicating mass transport limitation of this system. In order to examine the kinetic limitation of the proposed electrolyte system, much more aggressive current density of 10 mA cm^−2^ has been used and the results are shown in Supplementary Fig. S2. The ultra-high current density results in unstable and high voltage polarization (~120 mV) with irreversible drop after ~70h cycling in DX-DME/LiFSI electrolyte (Supplementary Fig. S2a). Correspondingly, the twining lumbricoid deposit can be observed on the granular lithium particles (Supplementary Fig. S2c). In comparison, the situation in EC-DMC/1M LiPF_6_ is worse. High voltage polarization up to 1 V and massive dendrites are noticeable (Supplementary Fig. S2b,d). Therefore, it is concluded that superior Li electrode performance can be maintained in DX-DME/LiFSI electrolyte with the current density of 5 mA cm^−2^, above which the kinetic limitation becomes apparent.

### Electrolyte’s compatibility with different cathodes

**Figure 6:**
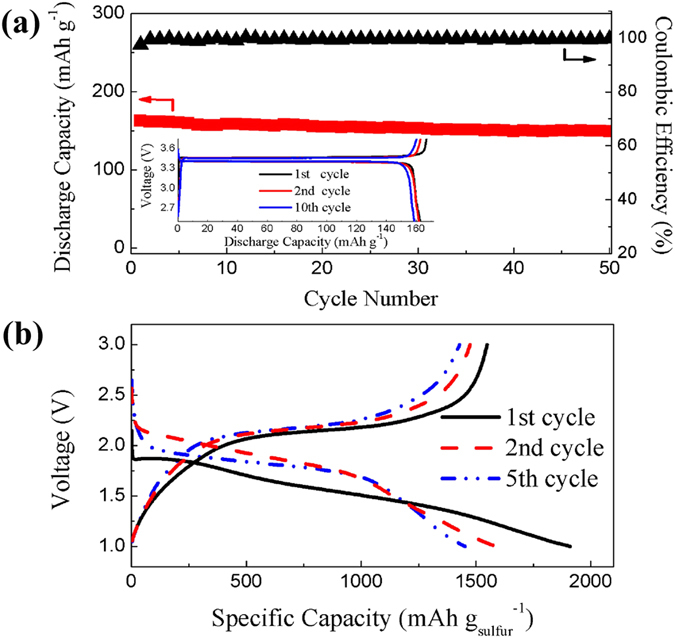
The compatibility of electrolyte with different cathodes. Cycling performance of (**a**) Li/LiFePO_4_ cell and Charge–discharge characteristics of (**b**) Li/S_composite_ cell with DX-DME/1M LiFSI electrolyte at 0.1C.

The compatibility of this ether-based electrolyte with cathode materials has been demonstrated in Li/LiFePO_4_ cell and Li/S_composite_ cell. The reversible cycle trends for both the cells in Fig. 6 confirm the good compatibility of this ether-based electrolyte with cathodes. A further development of this electrolyte may enable practical applications for rechargeable batteries based on lithium metal anode.

## Discussion

**Figure 7:**
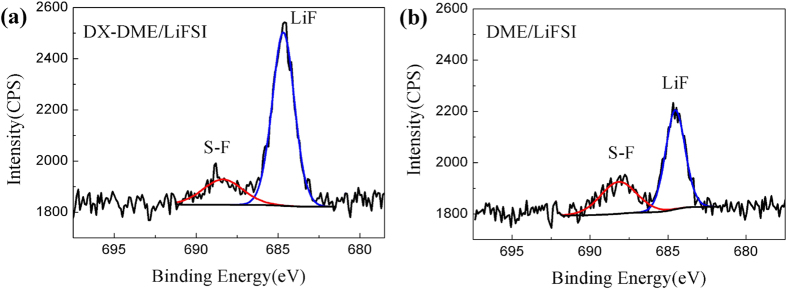
X-ray photoelectron spectroscopy (XPS) spectra of the F 1s regions of cycled Li surface. Lithium surface cycled in (**a**) DX-DME/LiFSI electrolyte and (**b**) DME/LiFSI electrolyte for 10 cycles at current density of 2 mA cm^−2^.

The extended anodic stability of this new ether-based electrolyte is largely associated with the introduction of DX as co-solvent. For one thing, the symmetric molecular structure with hexatomic ring in DX solvent could improve its anti-oxidation capability; For another, the coupling effects of solvent/salt would influence its stability as well. Based on the above two factors, wide electrochemical stability window up to ~4.87 V vs Li RE, the highest value to date for neat ether-based electrolyte system in lithium-metal based batteries, is obtained for this ether-based electrolyte. In addition to the good anti-oxidation capability of DX, its extremely low reduction potential of −1.95 V against Li/Li^+^ among various ethers (even lower than DME: −1.68 V), calculated according to *ab initio* DFT^25^, implies the low tendency to react with lithium, which will lead to a high coulombic efficiency. Particularly, when the less-reactive solvent is adopted, the composition of surface layer may be dominated by FSI^−^ anion reduction. It has been shown that the FSI-based ionic liquids could mitigate lithium dendrite formation as reported in previous research^32^. In order to better understand the electrolyte effect on the Li deposit structure, typical high-resolution region XPS spectra were taken to compare the composition of lithium surface cycled in electrolytes with and without DX. Supplementary Fig. S3 and Fig. 7 show a comparison of the C1s, Li1s, N1s, O1s, S2p and F1s spectra, which include the deconvolution of the broad peaks to specific peaks that reflect the various oxidation states of the elements and relevant peak assignments^3^,^33^,^34^,^35^,^36^. The highest peak of C1s here can be attributed to alkyl chain originated from solvent reduction products and has been calibrated to 284.8 eV. The XPS results shown in Fig. 7 indicate a clear and pronounced difference in F 1s spectra arising from the FSI^−^ anion reduction. Both F 1s spectra contain two peaks at 688.17 eV and 684.68 eV, which have been assigned to the presence of S-F (may be related to surface bound SO_2_F species) and LiF, respectively^35^,^36^. When DME is partly replaced by less reactive DX, anion reduction may become dominant. With DX-DME/LiFSI, stronger LiF response in Fig. 7a corresponds to more LiF formation in SEI layer. On the other hand, as shown in Supplementary Fig. S3, the surface components related to carbon, oxygen and lithium have no obvious difference for the two samples. But a slight difference could be found in the spectra of S2p and N1s. Two S2p peaks existed at 168.8 and 161.7 eV had been assigned to SO_2_F species and Li_2_S^34^,^36^, respectively, which may originate from the reduction of FSI^−^ anion. Weaker response of SO_2_F and N^−^ species and evidently stronger LiF response in Fig. 7 suggest the dominated LiF formation in SEI layer of Li electrode cycled in DX-DME/LiFSI electrolyte, which might facilitate a dense and higher conductive SEI layer and regulate the lithium deposit morphology, which leads to dendrite-free deposition and more stable voltage profiles even at high rate.

In addition, higher ionic conductivity and Li^+^ transference number (t_Li_^+^) of LiFSI compared to LiPF_6_ salt^37^ can improve the electrode kinetics and suppress lithium dendrite formation under high rate condition. According to Chazalviel *et al*. model, electrolytes with higher ionic conductivity and reduced anion mobility could suppress dendrite nucleation^32^,^38^.

In summary, a new DX-DME/LiFSI electrolyte system with extended anodic stability has been developed to address the low coulombic efficiency and dendrite formation during charge/discharge processes when using lithium metal as anode. In this electrolyte, DX solvent possesses not only low reactivity to lithium but also good anti-oxidation capability, under the effect of which a wide electrochemical stability window up to ~4.87 V vs Li RE, the highest value to date for neat ether-based electrolyte systems used in lithium-metal based batteries, has been achieved. The low chemical reactivity of this electrolyte and a more inorganic dense SEI layer result in very limited side reactions and thus a high coulombic efficiency (*ca.* ~98%) at full Li utilization. For the practical cells, the efficiency may be higher due to partial Li utilization. Furthermore, the superior Li cycling stability, non-dendritic lithium morphology at high current density as well as good compatibility of this electrolyte with cathodes favour its usage in lithium metal-based rechargeable batteries. This easy-to-use electrolyte offers insights into the understanding of electrolyte stability and open up a new approach to high energy-density rechargeable Li metal battery systems.

## Methods

### Materials

All the raw materials used in experiments are Li battery grade. LiFSI was obtained from Suzhou Fluolyte.Co.Ltd, which was firstly dried rigorously at 85 °C for 24 hours under vacuum. Anhydrous 1,3-dioxolane (DOL) and 1,2-dimethoxyethane (DME) were obtained from Aldrich, Inc. 1, 4-dioxane (DX) was obtained from Tokyo Chemical Industry (TCI), Inc. All the solvents were kept on 4 Å molecular sieves for at least 48 hours and subsequently stored in an Argon-filled glovebox, MBraun Labmaster. The electrolytes were prepared by dissolving the predetermined quantities of salt into the anhydrous solvents and the water content in the final DX-DME(1:2)/1M LiFSI electrolyte was 28.8 ppm measured by coulometric KF titrator (C30, Mettler Toledo). The separator used in coin cell is ENTEK ET 20-26 type (PE, thickness: 20 μm). The diameter of Li foil was 15.6 mm. The materials were stored and handled in an Ar-filled dry glove box containing less than 1 ppm water and O_2_.

### Electrochemical measurements

Coulombic efficiency and electrochemical cycling were measured using CR2016-type coin cells of a two-electrode configuration, which were assembled in the glove box containing less than 1 ppm water and O_2_. The coulombic efficiency of lithium electrode was examined using Li/electrolyte/stainless steel (SS) cells with ENTEK ET 20-26 (PE, thickness: 20 μm) used as separator and SS served as the substrate for Li metal deposition. To standardize the tests, 40 μl of electrolyte was used in each coin cell. During each cycle, a constant deposition current density of 0.25 mA cm^−2^ was passed through the cell for 2.5 h, and then the same dissolution current density was applied until the cut-off voltage of 1.2 V vs. Li. The processes of Li metal plating/stripping at current density of 0.5 mA cm^−2^, 2.0 mA cm^−2^, 3.0 mA cm^−2^, 5.0 mA cm^−2^ and 10.0 mA cm^−2^ were conducted using Li|Li symmetric cells, in which Li metal used as the working and counter electrodes.

Cyclic voltammograms were obtained in a three-electrode configuration inside the glove box using a CHI604A electrochemical work station (Shanghai, China). The working electrode was Pt disc electrode (area: 0.0314 cm^2^) and lithium slice served as counter electrode (CE) and reference electrode (RE). The Pt disc electrode was polished with Al_2_O_3_ powder and rinsed with alcohol before use.

Ionic conductivity measurements were carried out using a FE30 conductivity meter and an InLab 710 conductivity measuring cell (Mettler Toledo, Switzerland) by inserting the InLab 710 conductivity electrode in solution after calibration.

The LiFePO_4_ electrode and sulfur composite electrode^39^ with S content of 38.7% were fabricated by mixing active material, conductive carbon black (Super P, 40 nm, Timical) and binder in weight ratio of 80:10:10 for electrolyte compatibility measurement. The binder used in LiFePO_4_ electrode and sulfur composite electrode was poly(vinylidene difluoride) and cyclodextrin respectively. After casting the slurry on Al (for LiFePO_4_ with mass loading of ~8.2 mg cm^−2^) and Cu foil (for sulfur composite with mass loading of ~10.9 mg cm^−2^) current collectors, the electrodes were dried at 65 °C in vacuum for 4 h. The coin cells CR2016 were assembled with the above electrodes and 40 μl of electrolyte. The discharge and charge were measured at room temperature on a LAND-CT 2001A Battery Test System (Wuhan, China).

### Sample characterization

The morphologies of lithium metal electrodes were observed by FEI Nova Nano-scanning electron microscope (SEM). The Li metal discs were firstly harvested by disassembling the cycled coin cells and then thoroughly washed by anhydrous DME to remove any electrolyte salt residuals. To avoid exposure to air, the dried samples were sealed in an air-isolating container and transferred quickly into the SEM equipment under the protection of Ar flow. The X-ray photoelectron spectroscopy (XPS) analysis was performed using a Kratos Axis UltraDLD spectrometer (Kratos Analytical-A Shimadzu Group Company) with monochromatic Al Kα source (1486.6 eV). Before transferring the samples to the equipment, a specialized air-isolating container with protective Ar atmosphere was used to avoid moisture/air exposure. Under slot mode, the analysis area was 700 × 300 μm and analysis chamber pressure was less than 5 × 10^−9^ Torr. The binding energy was calibrated according to the C 1s peak (284.8 eV) of adventitious carbon on the analyzed sample surface.

## Additional Information

**How to cite this article**: Miao, R. *et al*. A new ether-based electrolyte for dendrite-free lithium-metal based rechargeable batteries. *Sci. Rep.* **6**, 21771; doi: 10.1038/srep21771 (2016).

## Supplementary Material

Supplementary Information
