# Supplementary material for: A new ether-based electrolyte for dendrite-free lithium-metal based rechargeable batteries
Source: Sci Rep. 2016 Feb 16;6:21771. doi: 10.1038/srep21771 (PMC4754942; doi:10.1038/srep21771)
Supplement: Supplementary Information [file srep21771-s1.pdf]

A new ether-based electrolyte for dendrite-free lithium-metal based rechargeable batteries

Rongrong Miao[a], Jun Yang*[a], Zhixin Xu[a], Jiulin Wang[a], Yanna Nuli[a] and Limin Sun[b]

[a] *School of Chemistry and Chemical Engineering, Shanghai Jiao Tong University, 800 Dongchuan Road, Shanghai, 200240, China*

[b] *Instrumental Analysis Center, Shanghai Jiao Tong University, Shanghai 200240, P. R. China.*

*To whom correspondence should be sent. E-mail: yangj723@sjtu.edu.cn

Supplementary Figures:

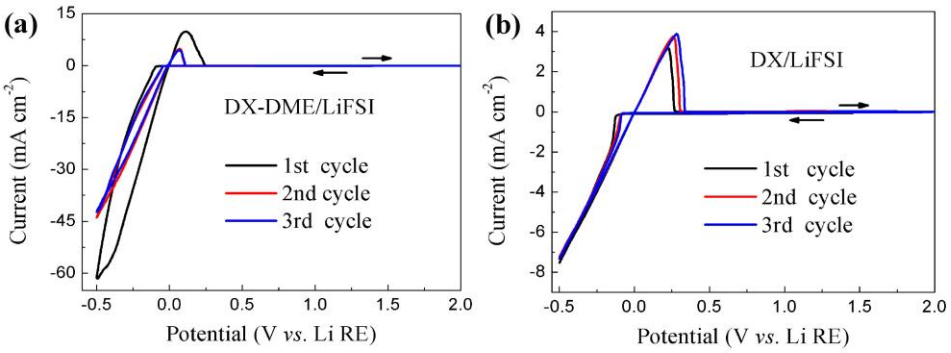

Supplementary Figure S1: Steady-state cyclic voltammograms on stainless steel (SS) electrode with different electrolytes at a scanning rate of 5 mV s^{-1}. (a) DX-DME/1M LiFSI; **(b)** DX/1M LiFSI

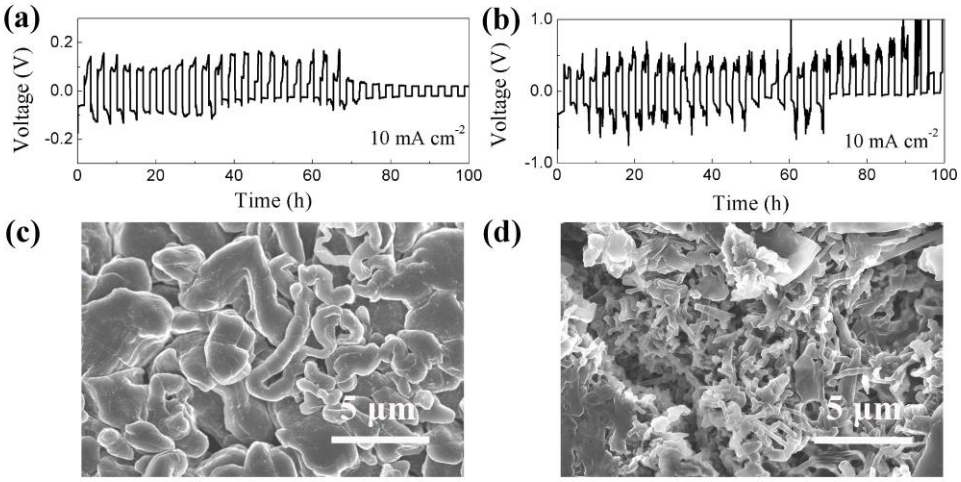

Supplementary Figure S2: Voltage versus time curves for a symmetric lithium cell under current density of 10 mA cm^{-2} and SEM analyses. Voltage profiles for **(a)** DX-DME(1:2)/1M LiFSI electrolyte and **(b)** EC-DMC/1M $LiPF_6$ electrolyte; SEM micrographs of deposited lithium after ~100h (30 cycles) in **(c)** DX-DME/1M LiFSI electrolyte and **(d)** EC-DMC/1M $LiPF_6$ electrolyte. The symmetric lithium cells cycled in above conditions last 1.7 h for each half-cycle and final state for SEM is deposited state.

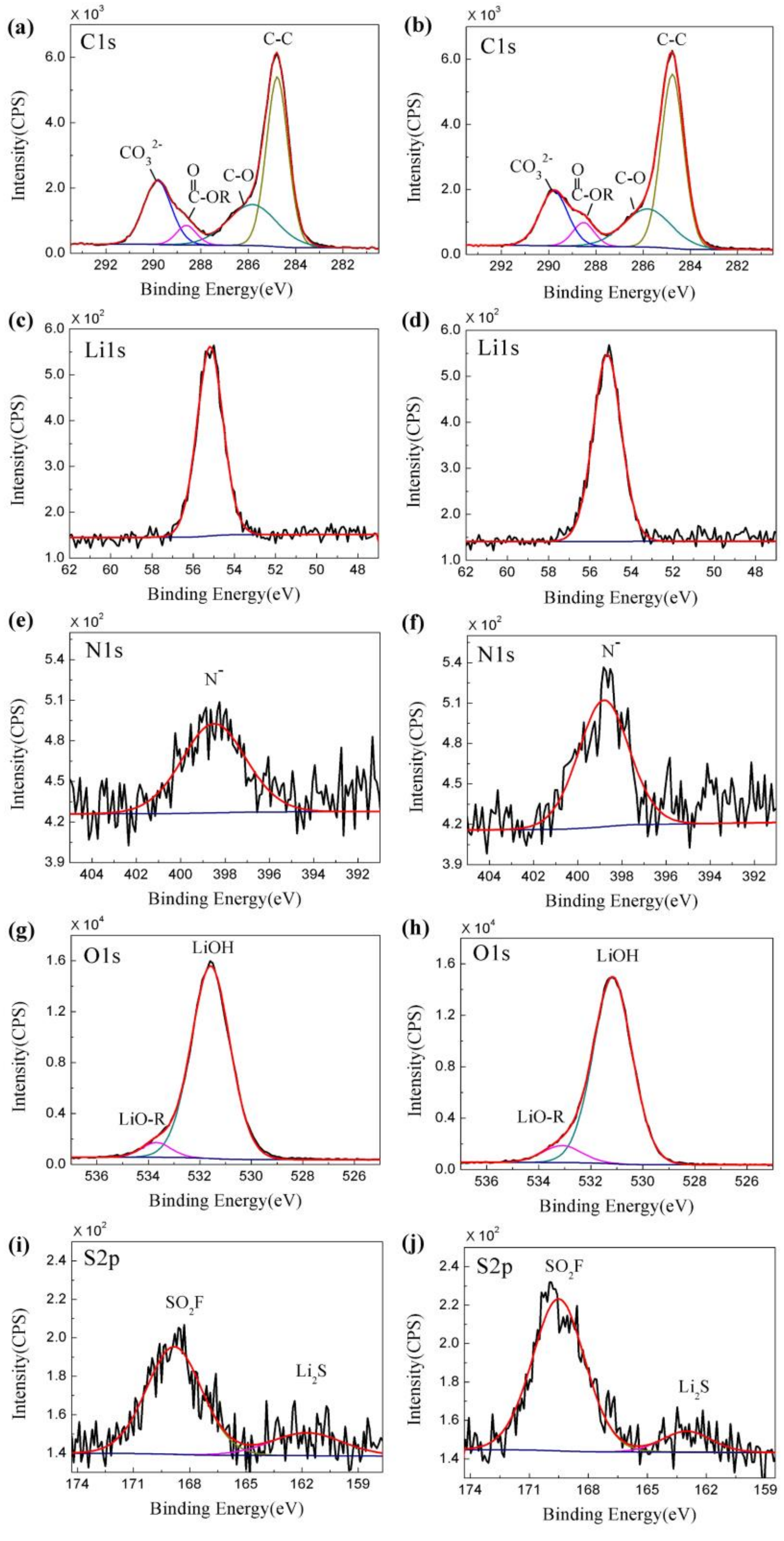

Supplementary Figure S3: XPS spectra (C1s, Li1s, N1s, O1s and S2p) measured from metallic lithium anodes after 10 cycles at a current density of 2 mA cm^{-2}. (a) C1s, **(c)** Li1s, **(e)** N1s, **(g)** O1s and **(i)** S2p belong to DX-DME/1M LiFSI electrolyte, and **(b)** C1s, **(d)** Li1s, **(f)** N1s, **(h)** O1s and **(i)** S2p belong to DME/1M LiFSI electrolyte.
